# The Use of Amalgams for Filling Teeth

**Published:** 1856-06

**Authors:** E. Osmond


### THE USE OF AMALGAMS FOR FILLING TEETH.

[An inaugural dissertation submitted to the Trustees and Faculty, of the Ohio College of Dental Surgery, for the degree of Doctor of Dental Surgery.]

BY E. OSMOND.

That one of the most beautiful, and useful structures of which our bodies are composed, and which is also the firmest and densest of them, should be so often doomed to early decay, leaving others still in the pride of youth, and beauty, to mourn their loss, is a matter of deep and serious regret. But, alas! how frequently do we observe, that ere our organism has reached a few years in its progress of development, that "The Teeth," those organs of usefulness, and beauty, and I may add too, of expression, are seized by the destroyer, and by degrees waste and decay; causing us to suffer frequently so much pain, that we are glad to have them removed per force from our agonizing frames.

It is, I believe, a generally admitted fact, that caries of the teeth, is caused by a chemical action, or force, acting as an exciting cause, upon a previously existing diathesis or predisposition; and the chemical force, and this diathesis, may be either local, or constitutional, or both.

Thus may we have the constitutional diathesis, by an imperfectly formed dentine, and a deficiency of the osseous material.

So also may we have a crowded condition of the dentures, or irregularity, or malformation of them, constituting a local predisposition.

These causes, the constitutional, and local, may and often do exist conjointly, and the former may even cause the latter, by producing an irregular development; as where the early decay of the temporary organs causes irregularity to occur in the arrangement of the permanent set.

The constitutional chemical forces, may be from a mercurial diathesis of the general system, influencing the salivary glands, and so causing a perverted secretion from them, or from other constitutional states of the general health, which cause an acrimonious, or viscid state of the buccal fluids; such as the effects of continued fever, a residence in a low flat country particularly if miasmatic, dyspepsia, scorbutus, and scrofula, which last one may act in two ways. In the first place, when inherited it predisposes the teeth to decay by influencing the progress of their formation, and in the second, when developed in after life, it so changes the condition of the fluids of the mouth as to produce a chemical action upon them, and so cause their destruction, and this is particularly observable in phthisis. A number of diseases, which it were tedious to mention, may change the normal condition of the buccal fluids, by their action on the general constitution.

The chemical causes, which are of a local nature, are those caused by the fermentation of articles of food in the mouth, producing acid; and those which retain the fluids of the mouth around the teeth, and so change their condition; among these may be enumerated, salivary calculus, particularly the soft sponge like variety; roots of teeth, and uncleanliness, also acids of any description, which either dissolve the phosphate of lime, or decompose it, by their affinity for lime, and strong alkalies, which dissolve the animal portion of the dentine, leaving the calcareous, and most powerful of all, galvanic action, which has the power of decomposing acid compounds, liberating the acid in a free state, to combine with the dentine, causing its destruction.

I have thus briefly adverted to the causes of decay of the teeth, as I shall allude to them in the course of this treatise.

As the dentures are composed in their bony structure, of two pricipal substances, enamel and dentine; the former inclosing the crown of the other, and when formed, are permanent in structure, and not subjected to those changes which the other osseous tissues of the body undergo, it is generally admitted that caries always begins externally, and after penetrating the enamel, proceeds internally. Its progress being influenced by the nature and amount of acid substances formed, or deposited, in the cavity, which soften and destroy the dentine, it is found that any substance, that is capable of resisting the acids of the mouth, and also possesses sufficient density and firmness to fill the cavity, thoroughly, and permanently, will if properly applied, arrest the caries of the particular cavity to which it is applied, care being taken to remove the decayed portion of dentine from the cavity.

A number of experiments have been tried to find the best material for filling teeth. Gold and platinum, silver and tin foil, are each used by their respective advocates. Gold, in foil and sponge, and crystalline form, is much used, especially foil.

Of an old mode of practice, which some of the dental profession are trying to revive, it is my purpose to treat, namely:

FILLING WITH AMALGAMS.

An amalgam may be defined, as a compound of any metal or metals, capable of combining with metallic mercury, united with it in any proportion, in a metallic state.

The common manner of manufacturing an amalgam for filling teeth is to take one, two, or more metals, such as tin, silver, cadmium, lead, etc., and after reducing them to a fine state of division, add as much mercury, as is sufficient to bring them to a liquid consistence, then press out the superabundant mercury, through buckskin, or other close material, then while soft, introduce it into the cavity, and in a short time it will become hard, and will apparently be a successful operation, but whether it is of benefit to the patient, I purpose to discuss.

Although successful at first sight, and apparently filling the cavity perfectly, and having a bright metallic lustre, it is found, that not only does it oxydize on its external surface, but that every portion of the internal surface of the filling becomes black, proving conclusively, that gases of some kind have been in contact with it. I shall next consider what gases are likely to be found under such conditions.

As when two metals, one of them having a greater affinity for oxygen, or a corroding agent, than the other, are brought into contact, and exposed to the action of fluid, possessed of even a very small degree of acidity, galvanic action is produced ; it follows, that in fillings of this kind, it must necessarily occur, and this will inevitably decompose any saline substance which may be present in solution in the fluids of the mouth.

Thus chloride of sodium, which is an article of dailyuse, and also exists in healthy saliva, will necessarily be decomposed by such action, and the chlorine being liberated, will, whilst in a nascent condition, unite with the mercury of the amalgam, and in consequence of its well known tendency to unite with it in such proportions as will form corrosive sublimate, dangerous constitutional symptoms may be produced in consequence of the solubility of that salt.

Now it is urged by some, that the mercury is in chemical combination with the other metals. Admitting this to be the case, it is also certain that loose mercury is present, waiting to be set free in the form of vapor, or to unite with any free acid in the mouth, for which it has an affinity, and so to become the positive pole of a galvanic battery.

Further, it is well known that all alloys have a greater tendency to oxydize than single metals, and amalgams possess this in a marked degree. For example: expose a looking glass, the back of which is coated with amalgam, to the action of gases in a damp atmosphere, and how soon will it be observed to loose its reflecting power, and become tarnished by streaks of oxyde between the surface of the glass and that of the amalgam next to it. How then can we expect it to resist the action of the saline fluids, and gases of the mouth? and is it any wonder, that the oxydation goes on as well upon its internal surface, as upon its external one?

Hence as the mercury is removed from the amalgam, the surface of the plug assumes a dark color, and as the action of the chlorine extends its influence along the borders of the cavity, it is slowly, and surely, according to the state of the buccal fluids, removed from the internal surface of the filling, leaving it also as black as the external surface, and the walls of the cavity exposed to the action of the retained and, consequently vitiated fluids of the mouth.

Now let us suppose the mercury all removed by the above process, and what have we left? A plug lessened in size, and acid fluids around it, the dentine has become the positive pole of the battery, and the plug the negative. As a consequence, the dentine is rapidly dissolved by the acid fluids contained between it and the plug.

In proof of this position, we have only to look at the fact that when a plug of any description, becomes loose, or porous, or from want of cleanliness, the surface of a tooth, in contact with a filling is suffered to retain acid fluids, in contact with both, galvanic action is produced, and the destruction of dentine frequently goes on more rapidly than before a filling was introduced.

Further the presence of a metal is not necessary to the production of galvanic action; for when two conducting bodies whose affinity for a corroding agent is different, are placed in contact, it is produced, and this seems to account for the great destruction of dentine, which is found beneath very minute holes in the enamel, and which is particularly observable in caries of the central depressions of the crowns of the molar teeth.

In such cases the dentine seems to be the positive pole, being more easily acted upon than the enamel, and in this manner protects the latter to some extent, from the action of the corroding agent.

Hitherto I have only alluded to the action of chlorine in a nascent condition, but when it has no substance to unite with in that condition, hydrochloric acid is produced at the expense of the water of the saliva, and dissolves the dentine around a plug with rapidity. We have also, the septic acid, acetic acid, and others, which are well known to posses the power destroying the integrity of the enamel and dentine when applied to them directly, or when liberated from salts which contain them.

It is contended by some, that an amalgam plug never shrinks, and that in certain cases it does not become loose. Well admitting this to be so, for argument sake, I shall proceed to show, that shrinkage in its common acceptation is not necessary to cause oxydation of the internal surface of a filling, for as the mercury finds its way out of it, it is left of a porous texture, more, or less, and consequently, as the ends of the minute pores through which the mercury passes, are presented to the walls of the cavity, it retains its position there until the destruction of the dentine around, causes it to become loose. This spongy texture is easily observed in the oxyde upon its internal surface.

It has been observed by many writers on chemistry, that a union of mercury with other metals augments their disposition to oxydize in a great degree, whilst its own is also greatly increased.

The well known tendency which mercury has to produce the condition of things known as ptyalism, and that chronic condition of disease of the buccal fluids, attendant on the mercurial diathesis, wherever that metal is taken into the system by any means, should be taken into consideration. It is a fact, which has been observed by the most prominent members of the dental profession, that where amalgam fillings have been used in certain constitutions, ptyalism has been the the result, exfoliation of the alveoli, inflammation of the entire buccal cavity, and of the tissues adjoining, proving beyond doubt, that mercury had been absorbed, and that the said mercury was absorbed from the fillings, was proven by the fact that upon the removal of them the symptoms ceased, and even in cases where those acute symptoms were not produced, an unhealthy state of the buccal fluids was produced, which destroyed the other teeth, and many cases have been observed, where in consequence of a single amalgam plug in the mouth, the whole of the dentures have been destroyed.

It has been said that teeth that are too far gone to bear the introduction of any other filling, can be filled with ease with amalgam. In such cases I would respectfully submit, that a tooth of a description, that can neither be filled with foil nor crystal gold is better "out of the mouth than in it," and that it is a bad practice to endanger the remaining dentures, by adding to the evils of a bad tooth, those of a worse filling; and the conscientious dentist would better satisfy his principles of honor, and sense of the dignity of his profession, by extracting the tooth, gratis, should his patient permit, than by filling it with amalgam, for either a small, or great price, to add to the contents of his pocket.

But some say it can be so easily introduced, and so rapidly. I hope no man amongst us wishes to be considered a mere mechanical operator, in this ninteenth century, and age of improvement. No, I am happy to say, that the members of the dental profession, generally aim to elevate its standard, and banish quackery from our ranks. In order then that we may be scientific men, instead of mere mechanical operators, we study anatomy, physiology, pathology, therapeutics, chemistry, and the institutes of our science, and consequently, should know the *modus operandi*, so far as is known, of every agent used in, or having a relation to our profession, whether beneficial, neutral, or injurious; and not only to the teeth but to the system in general, and knowing these things, we claim and hold a special rank, and relation to the general physician. Now what judicious physician, aware of the tendency of mercury, to let the brunt of the action fall upon the salivary glands, would place it in such close proximity to them, when possible to avoid it. For it appears to have as special an affinity for them, as arsenic has for the stomach, no matter in what part of the system it may be introduced. If I wished to produce the alterative effects of mercury upon a patient, and had no respect for his teeth, an amalgam plug would fulfil the intention admirably, constantly, and a little stronger than homœpathically, but otherwise, I should prefer not inserting one.

Truly this is, "putting an enemy in a man's mouth to steal away (not his brains), but his teeth."

The physician who so far forgets the interests of his patient as to prescribe a medicine to produce temporary relief, knowing that it will eventually augment the diseased action, for the sake of getting a reputation for quickness, and dexterity; and there are many who do so, justly should be banished from the profession, and from society. Will then the members of the young and rising dental profession, from whom the public expect so much, so far forget their obligations to their patients as to practice the like quackery?

